# Clinical Spectrum and Tumour Risk Analysis in Patients with Beckwith-Wiedemann Syndrome Due to *CDKN1C* Pathogenic Variants

**DOI:** 10.3390/cancers14153807

**Published:** 2022-08-05

**Authors:** Leila Cabral de Almeida Cardoso, Alejandro Parra, Cristina Ríos Gil, Pedro Arias, Natalia Gallego, Valeria Romanelli, Piranit Nik Kantaputra, Leonardo Lima, Juan Clinton Llerena Júnior, Claudia Arberas, Encarna Guillén-Navarro, Julián Nevado, Spanish OverGrowth Registry Initiative, Jair Tenorio-Castano, Pablo Lapunzina

**Affiliations:** 1 INGEMM-Instituto de Genética Médica y Molecular, Instituto de Investigación Sanitaria Hospital La Paz (IdiPAZ), Hospital Universitario La Paz, 28046 Madrid, Spain; 2 CIBERER, Centro de Investigación Biomédica en Red de Enfermedades Raras, ISCIII, 28046 Madrid, Spain; 3 ITHACA-European Reference Network, Hospital La Paz, 28046 Madrid, Spain; 4 Bionano Genomics, San Diego, CA 92121, USA; 5 Department of Orthodontics and Pediatric Dentistry, Chiang Mai University, Chiang Mai 50200, Thailand; 6 Instituto Fernandes Figueira IFF/FIOCRUZ, Rio de Janeiro 22250-020, Brazil; 7 Hospital de Niños Dr. Ricardo Gutiérrez, Sección Genética Médica Gallo 1330, C1425EFD CABA, Argentina; 8 Sección Genética Médica, Servicio de Pediatría, Hospital Clínico Universitario Virgen de la Arrixaca, IMIB-Arrixaca, Universidad de Murcia, El Palmar, 30120 Murcia, Spain; 9 Spanish OverGrowth Registry Initiative, 28046 Madrid, Spain

**Keywords:** Beckwith-Wiedemann, imprinting disorders, *CDKN1C*, overgrowth, massive parallel sequencing, neoplasia, methylation, tumour

## Abstract

**Simple Summary:**

Beckwith–Wiedemann syndrome (BWS) is an overgrowth disorder caused by imprinting or genetic alterations at the 11p15.5 locus. BWS is considered a spectrum disorder (BWSp) with an increased neoplasm incidence. *CDKN1C* variants have been reported in 5–10% of patients, with a higher incidence in familial cases. In this study, we examined the clinical and molecular features of all cases of BWSp identified by the Spanish Overgrowth Registry Initiative with *CDKN1C* variants, ascertained by Sanger sequencing or next-generation sequencing. We present 21 cases, 19 of which were classified as classical BWS and 1 that developed a mediastinal ganglioneuroma. Our study supports the high heterogeneity of the clinical features of BWSp and adds evidence on tumour development in this BWSp molecular subgroup. Genotype–phenotype correlation studies of patients with suspected BWS are essential for improving the diagnosis and assessing whether its cause can be directly related to the BWS clinical spectrum in the few cases that develop tumours.

**Abstract:**

Beckwith–Wiedemann syndrome spectrum (BWSp) is an overgrowth disorder caused by imprinting or genetic alterations at the 11p15.5 locus. Clinical features include overgrowth, macroglossia, neonatal hypoglycaemia, omphalocele, hemihyperplasia, cleft palate, and increased neoplasm incidence. The most common molecular defect observed is hypomethylation at the imprinting centre 2 (KCNQ1OT1:TSS DMR) in the maternal allele, which accounts for approximately 60% of cases, although *CDKN1C* pathogenic variants have been reported in 5–10% of patients, with a higher incidence in familial cases. In this study, we examined the clinical and molecular features of all cases of BWSp identified by the Spanish Overgrowth Registry Initiative with pathogenic or likely pathogenic *CDKN1C* variants, ascertained by Sanger sequencing or next-generation sequencing, with special focus on the neoplasm incidence, given that there is scarce knowledge of this feature in *CDKN1C*-associated BWSp. In total, we evaluated 21 cases of BWSp with *CDKN1C* variants; 19 were classified as classical BWS according to the BWSp scoring classification by Brioude et al. One of our patients developed a mediastinal ganglioneuroma. Our study adds evidence that tumour development in patients with BWSp and *CDKN1C* variants is infrequent, but it is extremely relevant to the patient’s follow-up and supports the high heterogeneity of BWSp clinical features associated with *CDKN1C* variants.

## 1. Introduction

Beckwith–Wiedemann syndrome (BWS, MIM#130650) is an overgrowth disorder with a wide spectrum of phenotypic manifestations that can be recognised starting in the prenatal period. Clinical findings can include neonatal hypoglycaemia, macroglossia, hemihyperplasia, earlobe abnormalities including creases/pits, abdominal wall defects (omphalocele, umbilical hernia, and diastasis recti), visceromegaly, adrenocortical cytomegaly, renal abnormalities (e.g., medullary dysplasia, nephrocalcinosis, medullary sponge kidney, and nephromegaly), cleft palate, and a higher predisposition to embryonic tumours compared with the general population [1,2,3]. Due to the highly heterogeneous clinical manifestations, BWS has been redefined as Beckwith–Wiedemann spectrum (BWSp) [2].

BWS is commonly diagnosed during the perinatal period or in early childhood, and there are no sex differences in the frequency of its onset, except for monozygotic twins, who show a notable female predominance [4]. The estimated prevalence of BWS is approximately 1 case per 12,000 live births, although it is higher for babies born from assisted reproductive techniques, in which the prevalence is increased to 1 per 1126 live births [5,6,7].

BWS is caused by epigenetic, genomic, and genetic alterations within the 11p15.5 locus, which contains a cluster of genes that participate in cell cycle regulation, proliferation, and somatic growth and that are organised into two separate imprinting centres. These imprinting centres are characterised by a differential methylation pattern of their maternal and paternal alleles [8,9,10]. Due to their complexity, BWS cases have been divided into five molecular subgroups: (1) gain of methylation (GOM) at imprinting centre 1, which includes *H19* and *IGF2*, also referred to as the H19/IGF2 intergenic differential methylated region (H19/IGF2:IG DMR); (2) loss of methylation (LOM) at imprinting centre 2, which encompasses *KCNQ1OT1* and *CDKN1C*, also known as the KCNQ1OT1 transcriptional start site differential methylated region (KCNQ1OT1:TSS DMR); (3) segmental mosaic paternal uniparental disomy (UPD(11)pat); (4) deletions/duplications at the 11p15.5 locus; and (5) pathogenic *CDKN1C* variants [4,11,12].

DNA methylation abnormalities are the most frequent abnormality of the 11p15 region, with approximately 50–60% of patients presenting with LOM of KCNQ1OT1:TSS DMR and 5–10% presenting GOM of H19/IGF2:IG DMR [13,14]. In approximately 20% of cases, segmental mosaic UPD(11)pat has been identified, and fewer than 5% of cases are due to chromosomal abnormalities at 11p15 [15,16]. Additionally, 5–10% of sporadic and 40% of familial BWSp cases have intragenic *CDKN1C* pathogenic variants [17,18], and *CDKN1C* variants have also been observed in women with preeclampsia/HELLP (haemolysis, elevated liver enzymes, and low platelets) who were mothers of patients with BWS [19]. Unfortunately, the molecular diagnosis is still inconclusive in up to 10–15% of cases of clinically diagnosed BWSp [2,20], suggesting that other genes/loci can contribute to the clinical spectrum manifestations of BWS.

Embryonic tumours are detected in approximately 8% of children with BWSp, the most frequent being Wilms tumour (52%), followed by hepatoblastoma (14%), neuroblastoma (10%), rhabdomyosarcoma (5%), and adrenal carcinoma (3%) [21]. The highest tumour risk has been associated with GOM of H19/IGF2:IG-DMR (28% of cases) followed by UPD(11)pat (16% of cases). KCNQ1OT1:TSS-DMR LOM and *CDKN1C* intergenic variations account for fewer than 7% of BWS cases with embryonic tumours [21]. The tumour types observed vary according to the BWSp molecular subgroups: cases with H19/IGF2:IG DMR GOM are usually predisposed to Wilms tumour, patients with KCNQ1OT1:TSS DMR LOM or *CDKN1C* pathogenic variants are predisposed to hepatoblastoma, rhabdomyosarcoma, or neuroblastoma, and patients with UPD(11)pat are predisposed to any of the tumour types observed in the BWS spectrum [2,10,12,15,21,22].

The overall BWSp tumour risk is highest in the first two years of life and appears to reduce progressively until puberty, at which point there is almost the same cancer risk as for the general population [2]. Given that tumour surveillance varies according to regional medical practices and local healthcare systems, the BWSp International Consensus Group [2] agreed that tumour surveillance should focus on those molecular subgroups of BWSp that are at highest risk, and that patients with BWSp with a molecular diagnosis within the BWSp subgroups and patients with diagnosed classical BWS and no detectable molecular abnormality should be offered abdominal ultrasound every 3 months until the age of 7 years. Most European countries also suggest that patients with BWSp undergo hepatoblastoma screening through serum alpha-fetoprotein measurements.

Previous genotype–phenotype studies from our group, in collaboration with the Spanish Overgrowth Syndromes Registry Initiative (SOGRI), have described the phenotypic differences between cases of BWSp and the tumour risk involved. In a series of 72 patients with BWSp [23], we identified 8 individuals with *CDKN1C* pathogenic variants and subsequently reported [24] a pathogenic *CDKN1C* variant that segregated in a family presenting BWSp. In this study, we report on 10 additional patients with BWSp and *CDKN1C* variants and review the clinical spectrum of all of these previous cases (for a total of 21 cases of BWSp), with the aim of improving the genotype–phenotype correlations and calculating the tumour risk. All cases were analysed either by classical Sanger sequencing or by massive parallel sequencing.

## 2. Materials and Methods

**Patients**: All patients were selected from the SOGRI Consortium and met the clinical criteria of BWSp according to the scoring classification proposed by the Consortium [2]. All patients or their legal tutors gave their informed consent for study participation, and the project was approved by the Ethics Committee for Scientific Research of La Paz University Hospital (CEIm PI-1543).

**Molecularstudies:** All patients were tested for chromosomal abnormalities and epigenetics alterations in the 11p15 locus by karyotype, SNP-array, and methylation-specific multiplex ligation-dependent probe amplification. Patients were analysed by either Sanger sequencing or by a custom next-generation sequencing (NGS) panel. For those analysed by Sanger sequencing, specific primers were designed to amplify the two exons of *CDKN1C*, including the exon–intron boundaries (primers available upon request). *CDKN1C* analysis by NGS was performed using a custom panel of 236 genes designed in-house (Overgrowth v3.1). Library preparation was performed with the Kapa Library (Roche, CA, USA) following the manufacturer’s instructions, and sequencing was performed with Illumina NextSeq500 equipment. Bioinformatic analysis and variant prioritisation was performed with an in-house custom algorithm, including copy number variation detection (LACONv, https://github.com/kibanez/LACONv, accessed on 9 December 2021). Segregation analysis was performed in parental samples by Sanger sequencing when samples were available. Variants were described using Human Genome Variation Society nomenclature and classified according to the American College of Medical Genetics guidelines [25], adapting the imprinting pattern of inheritance, not initially contemplated in these guidelines.

## 3. Results

We analysed *CDKN1C* variants in 10 novel patients with BWSp and identified six previously undescribed pathogenic variants. We also reviewed 11 BWSp cases with *CDKN1C* variants previously reported in collaboration with the SOGRI initiative [23,24], reaching 21 cases of BWSp from 15 unrelated families. None of these patients presented chromosomal abnormalities, microdeletions, microduplications, or epigenetics (methylation) abnormalities in the 11p15 locus.

From the cases described for the first time in this study, eight were analysed by Sanger sequencing, while the two remaining (corresponding to a proband and their sibling) were analysed by the NGS gene panel. Segregation analysis confirmed a maternal inheritance in all 10 cases. Four out of the six novel variants identified were missense, and the two other variants corresponded to a loss-of-function and an intronic variant. All variants identified are located at exon 1 of *CDKN1C*, were absent in the analysed pseudocontrol population databases, and the majority of the in silico pathogenicity predictors applied suggested a deleterious effect (Table 1). The previously reported cases included nine *CDKN1C* variants, with a total of 15 *CDKN1C* variants in 21 cases of BWSp, from 15 unrelated families (Table 1).

**Figure 1:**
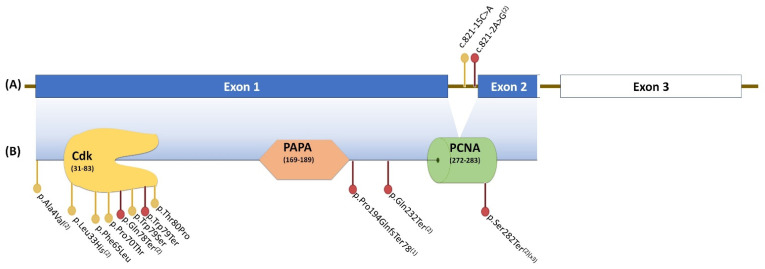
Variants identified along the *CDKN1C* gene, organised in three exons (**A**). Exons 1 and 2 encode the functional protein (**B**), which is organised into three domains: the cyclin-dependent kinase-inhibitor domain (CdK), the proline–alanine repeats domain (PAPA), and the proliferating cell nuclear antigen domain (PCNA). ^(1)^ Variants reported by Kantaputra et al. (2013) [24]; ^(2)^ Variants reported by Romanelli et al. (2010) [23]; ^(2)(X3)^ Variants reported by Romanelli et al. (2010) that were observed in three different cases. Red dots, loss-of-function variants; yellow dots, missense variants.

The reported patients included a family we previously described [24], whose two daughters and a son shared a maternally inherited likely pathogenic frameshift variant in exon 1 of *CDKN1C* (Table 1). The other eight cases [23] corresponded to patients with classic or isolated BWSp, in which both missense and loss-of-function variants were identified in exons 1 and 2 of *CDKN1C* and whose family segregation studies were able to identify one de novo patient and the maternal inheritance of the variants in another six individuals (Table 1). Figure 1 shows the 15 variants identified along the *CDKN1C* gene.

Clinical data were reviewed for all 21 cases, all of them with cardinal and/or very suggestive features of BWS (Table 2). Macroglossia was observed in all cases. Abdominal wall defects, which include omphalocele and umbilical hernia, were observed in 16 (76.2%) patients, with two cases having both features. Other common clinical features included posterior helix pits (38.1%), anterior earlobe creases (38.1%), transient hypoglycaemia (38.1%), cleft palate (33.3%), overgrowth (33.3%), nevus flammeus (23.8%), and inguinal hernia (18%). Six cases presented premature birth (28.6%), ranging from 29 to 36 weeks. Hemihypertrophy, thin upper lip vermilion, coarse facial features, and anterior open-bite malocclusion each accounted for 14.3% of cases. Uncommon clinical features, observed in at least two patients, included capillary malformation, hepatomegaly, postaxial foot polydactyly, premature tooth eruption, sensorineural hearing impairment and supernumerary flexion creases of the fingers. Macrocephaly, craniosynostosis, hypotonia, cryptorchidism, hypospadias, thyroglossal cyst, renal cyst, nephromegaly, splenomegaly, haemangioma, prominent upper eyelids, strabismus, apnoea, and psoriasiform dermatitis accounted in less of two patients.

The clinical features were analysed according to the BWSp consensus scoring proposed by Brioude et al. [2] (Table 3). All patients except one fulfilled the classical BWS clinical criteria, with a BWSp score ≥ 4. The OGS1774 patient, the brother of OGS1380 from family 2, presented a score of 3 due to the presence of one cardinal BWSp feature (macroglossia) and one suggestive feature (transient hypoglycaemia).

The patients’ ages ranged from 9 to 45 years (Table 1), and most were adolescents or young adults. All of these patients were periodically followed-up for at least 7 years to evaluate the risk of tumour development. Only one patient (OGS1493) developed a mediastinal ganglioneuroma (at 21 years of age) (Table 2).

## 4. Discussion

BWSp presents a heterogeneous phenotype that comprises a wide clinical spectrum and a complex molecular aetiology. Therefore, a number of patients did not meet the classic clinical criteria for a BWSp diagnosis. Molecular diagnosis is strongly recommended to confirm the clinical suspicions, especially in those cases that do not meet all criteria suggested by the International Consensus Group [2]. Molecular confirmation is also useful for patient management because certain molecular subtypes present a higher tumour risk than others.

In terms of the clinical and molecular features of BWSp that we have studied over the past decade, we present a review of all 21 cases with pathogenic or likely pathogenic *CDKN1C* variants, in which we were able to re-analyse clinical features according to a BWSp score and the follow-up of these patients in terms of tumour development. From these 21 cases, we detected four missenses, one loss-of-function and one intronic *CDKN1C* variant, all maternally inherited in 10 novel individuals. In silico pathogenicity predictors suggested deleterious effects, and all of these variants were absent in several pseudocontrol population databases (Table 1), which led to the suggestion that these variants are causative.

From our previously reported remaining variants, most corresponded to truncating or frameshift variants distributed along the coding sequence of *CDKN1C*, whereas two variants were located within intron 1 (Figure 1). All missense variants, except from p.Ala4Val, were observed in the CdK domain, in agreement with the finding that most missense variants related to BWSp occur in this domain, leading to a possible loss of cell cycle inhibition and aberrant growth [26].

Patients with BWSp and *CDKN1C* variants usually present with abdominal wall defects, higher probability of cleft palates, genital abnormalities, polydactyly, or extra nipples, which helps distinguish this patient subgroup from those with epigenetic abnormalities [10,27,28]. Abdominal wall defects together with cleft palates were observed in six (28.6%) cases, all of them with loss-of-function *CDKN1C* variants, including the family described by Kantaputra et al. [24] and three patients previously reported by Romanelli [23], two of whom had nonsense variants in nucleotide c. 845. None of the 10 patients described for the first time in this study showed cleft palate, and the combination of abdominal wall defects with genital abnormality or extra nipples was not observed. In contrast, omphalocele together with anterior earlobe creases were observed in three of these 10 patients, from two distinct families: patient OGS2051 (family 5), who also presented with postaxial foot polydactyly, had a nonsense variant in nucleotide c.237 (p.Trp79Ter). Interestingly, the index case (OGS2052) and his sibling (OGS2166), from family 6, presented a missense variant at a close nucleotide position, c.238 (p.Thr80Pro).

All 21 patients presented macroglossia, a cardinal feature in BWSp despite the BWS molecular subtype [3,29]. According to the BWSp consensus scoring [3], 20 of these patients were diagnosed with classical BWS (score ≥4), and one was considered to have clinical features suggestive of BWS (score 3) due to the presence of macroglossia and transient hypoglycaemia: patient OGS1774 from family 2, carrier of the missense variant p.Phe65Leu, while his sibling, OGS1380, with a score 4, presented with generalised overgrowth and visceromegaly, in addition to the same clinical features as his sibling. The fact that both siblings have the same genetic variant and share macroglossia and transient hypoglycaemia might be due to the intrafamilial phenotypic heterogeneity that could be observed between patients who share the same pathogenic variant. It is important to note that some of the cardinal BWS features, such as hyperinsulinism and Wilms tumour, occur more frequently in patients with imprinting alterations at 11p15, usually in the molecular subgroups of segmental mosaic UPD(11)pat and of GOM of H19/IGF2:IG DMR, respectively. It is therefore likely that patients with imprinting abnormalities present a higher mean BWSp score compared with cases with pathogenic *CDKN1C* variants. Patients with *CDKN1C* have a higher frequency of exomphalos compared with other molecular subgroups and can have a variety of suggestive features. It is therefore highly difficult to establish whether there could be a difference in the mean BWSp score between cases with pathogenic *CDKN1C* variants and patients with imprinting abnormality. Similarly, we observed asymmetric regional body overgrowth (formerly hemihypertrophy/hemihyperplasia) [30] in 3/21 (14%) patients. Asymmetric regional body overgrowth is infrequently observed in patients with BWSp with *CDKN1C* pathogenic changes. The same proportion has been found in a large series of 57 individuals with BWSp due to pathogenic/likely pathogenic *CDKN1C* variants (7/57, 14%) [31].

Several groups have reported uncommon clinical features in BWSp cases with *CDKN1C* variants, which makes the clinical diagnosis of this group even more challenging. Jukiewics et al. (2020) [32] reported a pathogenic frameshift variant between Cdk and PAPA domains of *CDKN1C* (p.Pro140Serfs*133) in a patient with sporadic BWSp and supernumerary flexion creases. This clinical feature was not observed in the 10 patients described for the first time in this study, although it was observed in a family we previously reported on [24] in which there was also a frameshift variant (p.Pro194GlnfsTer78) between the PAPA and PCNA domains. Therefore, in agreement with Jukiewics et al. (2020), we suggest that BWS cases with *CDKN1C* variants should be clinically reevaluated to assess whether supernumerary flexion creases could be a clinical feature for patients with BWS with loss-of-function *CDKN1C* variants. Further studies are needed to evaluate the impact of these frameshift variants, which truncate at least the PCNA domain of the protein, to improve their possible genotype–phenotype association.

Given that BWSp is caused by epigenetic/genetic alteration in gene clusters involved in cell cycle progression and growth control, tumour risk represents an important clinical feature and a concern in patient management. Although it is difficult to precisely estimate, varying tumour risks are associated with BWS molecular subgroups: patients with H19/IGF2:IG DMR GOM have the highest risk (28% risk), followed by those with segmental UPD(11)pat (16% risk), loss-of-function *CDKN1C* variants (6.9% risk), and lastly, KCNQ1OT1:TSS DMR LOM cases (2.6% risk) [21]. Most tumours occur in the first 6 years of life, although in certain cases, tumours are diagnosed after this age, such as a 10-year-old patient with a loss-of-function *CDKN1C* variant who developed T-type acute lymphoblastic leukaemia [33,34]. As proposed by Brioude et al. [34], the difficulty in precisely estimating the tumour risk in BWSp cases with *CDKN1C* variants is due to the scarcely reported tumour cases and the onset of tumour types not usually observed in this patient group [10,18].

Some of the significant features of BWSp, such as macroglossia and postnatal overgrowth, tend to ameliorate over the years. Patients with BWSp might therefore have irregular follow-ups during adulthood unless they have been diagnosed in childhood [3]. This can lead to an underestimation of BWSp cases, which can influence the risk of developing neoplasms. Another factor that might affect the precise estimation of the tumour risk in patients with BWSp and *CDKN1C* variants could be related to the lack of diagnoses during adulthood or clinical management until their adolescent/early adult life. Gazzin et al. [35] reported the tumour incidence in a series of 26 adult cases with molecular diagnosis of BWSp, eight of whom developed tumours. All of them showed epigenetic alterations, except for one case with a *CDKN1C* variant who developed intratubular germ cell neoplasia at 27 years of age; although, as proposed by the authors, these data could be overestimated because of the study design, and other factors that might lead to tumour development could not be ruled out. Nevertheless, a few other cases with pathogenic *CDKN1C* variants have been diagnosed in adulthood with BWSp and cancer: a mesoblastic nephroma in a 33-year-old male patient [12] and a superficial spreading melanoma that was diagnosed in a 42-year-old female patient [18]. Other patients with BWSp and neoplasms have been reported: congenital alveolar rhabdomyosarcoma during the neonatal period [36], neuroblastoma [17,18,21], ganglioneuroma [18], acute lymphoblastic leukaemia [18], and melanoma [18], among others. Lastly, a patient with a single-nucleotide *CDKN1C* variant and a clinical phenotype of IMAGe (intrauterine growth restriction, metaphyseal dysplasia, adrenal hypoplasia congenita, and genitourinary abnormalities syndrome, MIM#614732; the opposite phenotype of BWSp) with a rhabdomyosarcoma was recently reported [37].

In our series, one of the 21 (5%) patients developed a mediastinal ganglioneuroma at 21 years of age (patient OGS1493). This tumour rate in patients with BWSp with *CDKN1C* abnormalities is similar to rates reported in previous series [2,3,18,21,38]. Patient OGS1493 and his sisters had a loss-of-function *CDKN1C* variant located in the PAPA domain (Figure 1). These siblings shared many characteristics of BWSp, such as macroglossia, cleft palate, and omphalocele; however, sensorineural hearing loss, a less common feature, was observed in the proband and in his younger sister, once again supporting the intrafamilial phenotypic heterogeneity observed in patients that share the same pathogenic *CDKN1C* variant. The odds ratio of neuroblastic tumours in patients with *CDKN1C* mutations was calculated as close to 7 [10].

Very few patients with BWSp had developed neural crest tumours, with most of them located in the thorax and encompassing neuroblastomas, ganglioneuroblastomas and ganglioneuromas [9,39,40,41]. To our knowledge, thoracic ganglioneuromas have been reported in only two patients, both 4-year-old girls at the time of the diagnosis [39,41]. Unfortunately, the BWSp molecular group of these two patients was not reported. Although there is a relatively high prevalence of this tumour type in patients with *CDKN1C* variants [10], we cannot be certain that this neoplasm is directly linked to the loss-of-function *CDKN1C* variant or how this kind of variant can somehow affect tumour predisposition. Therefore, the phenotypic and molecular characterisation of other patients with BWSp who have developed some type of neoplasm, mainly from the neural crest, and functional studies of loss-of-function *CDKN1C* variants on neural crest tumour development are important to better understanding the relationship between loss-of-function *CDKN1C* variants and these tumour types. It is important to note that neuroblastoma, the neural crest tumour type more frequently observed in BWSp, is usually diagnosed in individuals younger than 10 years, with the age at diagnosis being very important for clinical management [42]. The use of urinary tumour markers, such as vanillylmandelic acid and homovanillic acid and/or the catecholamine-to-creatinine ratio, combined with ultrasound scans, can help diagnose neuroblastoma. According to Brioude et al. (2018), however, there is currently no evidence that this kind of screening could improve the diagnosis, treatment, and survival of individuals with BWSp and neuroblastoma. The guidelines proposed by Kamihara et al. (2017) recommend neuroblastoma screening for patients with *CDKN1C* variants using abdominal ultrasound, urine vanillylmandelic acid, and homovanillic acid tests with chest X-rays every 3 months until 6 years of age, then every 6 months until 10 years. No screening is recommended for patients older than 10 years. However, these tests can also help diagnose other types of less invasive and malignant neural crest tumours that could be present mainly in patients with BWSp and the loss-of-function *CDKN1C* submolecular type. Given that there have been very few reported cases of adolescents/adults in whom the correlation between neoplasm and BWSp is unclear, screening beyond the current guidelines might be warranted. However, more data and large cohorts are needed to clarify this issue. Due to the rarity of crest tumours in BWSp, periodic chest radiography is not recommended; however, physicians should consider the possible emergence of this tumour type and maintain a strict follow-up for children and adult patients.

This study adds evidence of the incidence of tumours in patients with BWSp caused by pathogenic *CDKN1C* variants, which has an important consequence in clinical management, follow-up, and genetic counselling. It is important to remember that these patients should be followed up by specialists, and the tumour risk should be ruled out according to published guidelines [2,42] to standardise tumour surveillance protocols. Nevertheless, it is important to consider the possibility of tumour development during adolescence or early adulthood in certain cases. Our results might be skewed by the maternally inherited nature of most of the variants; more studies are therefore needed, especially in sporadic patients with *CDKN1C* variants.

## 5. Conclusions

In this study, we reported on 21 patients with BWSp with pathogenic *CDKN1C* variants, adding new previously unreported variants and expanding the clinical phenotype of this disease. Genetic counselling and follow-up of individuals with pathogenic *CDK1NC* variants should be performed carefully, considering the differences in tumour risk between the various aetiologies associated with BWSp and the rarity of the tumours described. For patients with BWSp in whom the clinical features do not meet the threshold for classification as classical BWSp, genetic screening is still essential to confirm or discard the clinical suspicion, given that patients with a milder form of the disease can present a pathogenic *CDKN1C* variant. Genotype–phenotype correlation studies in patients with suspected BWS, in children and in adolescents/young adults, are essential to not only improve the diagnosis of BWSp but also to assess whether, in the few cases that develop tumours, its cause can be directly related to the BWS clinical spectrum.

## Figures and Tables

**Table 1:** Variants detected in *CDKN1C*.

Patient	Age *	Family	Relative	Genomic Coordinate (hg19)	Variant	Exon/ Intron	Variant Type	Inheritance	Allele Frequency ^†^	Pathogenicity Predictors ^‡^	CADD	ACMG Prediction ^§^	Detection Method	Reference
OGS1360	13	1	Pr	chr11:2906484	*CDKN1C*(NM_000076.2):c.236G>C,p.(Trp79Ser)	1	missense	maternal	-	24/26	28.39	VUS	SS	This study
OGS1810	10	1	S	chr11:2906484	*CDKN1C*(NM_000076.2):c.236G>C,p.(Trp79Ser)	1	missense	maternal	-	24/26	28.39	VUS	SS	This study
OGS1380	12	2	Pr	chr11:2906527	*CDKN1C*(NM_000076.2):c.193T>C,p.(Phe65Leu)	1	missense	maternal	-	23/26	28.5	VUS	SS	This study
OGS1774	9	2	B	chr11:2906527	*CDKN1C*(NM_000076.2):c.193T>C,p.(Phe65Leu)	1	missense	maternal	-	23/26	28.5	VUS	SS	This study
OGS1449	12	3	Pr	chr11:2906512	*CDKN1C*(NM_000076.2):c.208C>A,p.(Pro70Thr)	1	missense	maternal	-	24/26	25.6	VUS	SS	This study
OGS1584	20	4	S	chr11:2905379	*CDKN1C*(NM_000076.2):c.821-15C>A	intron 1	Non-coding	maternal	-	1/2	19.38	VUS	SS	This study
OGS1775	16	4	Pr	chr11:2905379	*CDKN1C*(NM_000076.2):c.821-15C>A	intron 1	Non-coding	maternal	-	1/2	19.38	VUS	SS	This study
OGS2051	18	5	Pr	chr11:2906483	*CDKN1C*(NM_000076.2):c.237G>A,p.(Trp79Ter)	1	nonsense	maternal	-	8/9	37	P	SS	This study
OGS2052	12	6	Pr	chr11:2906482	*CDKN1C*(NM_000076.2):c.238A>C,p.(Thr80Pro)	1	missense	maternal	-	ago-26	23.7	VUS	GP	This study
OGS2166	17	6	S	chr11:2906482	*CDKN1C*(NM_000076.2):c.238A>C,p.(Thr80Pro)	1	missense	maternal	-	8/26	23.7	VUS	GP	This study
OGS1492	32	7	Pr	chr11:2906141	*CDKN1C*(NM_000076.2):c.579del,p.(Pro194GlnfsTer78)	1	frameshift	maternal	-	-	-	LP	SS	[24]
OGS1493	23	7	B	chr11:2906141	*CDKN1C*(NM_000076.2):c.579del,p.(Pro194GlnfsTer78)	1	frameshift	maternal	-	-	-	LP	SS	[24]
OGS1494	28	7	S	chr11:2906141	*CDKN1C*(NM_000076.2):c.579del,p.(Pro194GlnfsTer78)	1	frameshift	maternal	-	-	-	LP	SS	[24]
OGS1074	14	8	Pr	chr11:2906709	*CDKN1C*(NM_000076.2):c.11C>T,p.(Ala4Val)	1	missense	maternal	1/36.514	5/24	14.67	VUS	GP	[23]
OGS542	16	9	Pr	chr11:2906622	*CDKN1C*(NM_000076.2):c.98T>A,p.(Leu33His)	1	missense	maternal	-	8/10	26.4	VUS	SS	[23]
OGS4	24	10	Pr	chr11:2906488	*CDKN1C*(NM_000076.2):c.232C>T,p.(Gln78Ter)	1	nonsense	de novo	-	7/9	36	P	SS	[23]
OGS851	15	11	Pr	chr11:2906026	*CDKN1C*(NM_000076.2):c.694C>T,p.(Gln232Ter)	1	nonsense	maternal	-	5/9	37	LP	SS	[23]
OGS145	19	12	Pr	chr11:2905366	*CDKN1C*(NM_000076.2):c.821-2A>G	intron 1	splicing	maternal	-	7/9	33	P	SS	[23]
OGS1234	14	13	Pr	chr11:2905340	*CDKN1C*(NM_000076.2):c.845delC,p.(Ser282Ter)	2	nonsense	maternal	-	-	-	LP	SS	[23]
OGS491	20	14	Pr	chr11:2905340	*CDKN1C*(NM_000076.2):c.845C>A,p.(Ser282Ter)	2	nonsense	maternal	-	7/23	37	P	SS	[23]
OGS1356	45	15	Pr	chr11:2905340	*CDKN1C*(NM_000076.2):c.845C>G,p.(Ser282Ter)	2	nonsense	N/E	-	7/23	36	LP	SS	[23]

Pr, Proband; S, Sister; B, Brother; N/E, Not Evaluated; VUS, Variant of Uncertain Significance; P, Pathogenic; LP, Likely Pathogenic; SS, Sanger Sequencing; GP, Gene Panel. * Age in years; ^†^ Allele frequency was estimated from several population pseudo-control databases: gnomAD genomes (v3.0), gnomAD exomes (v3.1), Kaviar (version 160204-Public), Beacon (v2.0), 1000 G, Phase III, and Bravo (TOVMed Freeze 8); ^‡^ Analysis of several bioinformatic tools included in the dbNSFP (v3.0) database plus the computation of the CADD score (v1.6); ^§^ ACMG, American College of Medical Genetics.

**Table 2:** Clinical Features Observed in 21 Patients with BWSp.

Clinical Features		No. Patients	% Patients	
Macroglossia	HP:0000158	21	100.0	
Omphalocele	HP:0001539	13	61.9	
Posterior helix pits	HP0008523	8	38.1	
Anterior earlobe creases	HP:0009908	8	38.1	
Transient hypoglycaemia	HP:0001998	8	38.1	
Cleft palate	HP:0000175	7	33.3	
Overgrowth	HP:0001548	7	33.3	
Premature birth	HP:0001622	6	28.6	(ranging from 29 to 36 weeks)
Umbilical hernia	HP:0001537	5	23.8	
Nevus flammeus	HP:0001052	5	23.8	
Inguinal hernia	HP:0000023	4	19.0	
Hemihypertrophy	HP:0001528	3	14.3	
Thin upper lip vermilion	HP:0000219	3	14.3	
Coarse facial features	HP:0000280	3	14.3	
Anterior open-bite malocclusion	HP:0009102	3	14.3	
Capillary malformation	HP:0025104	2	9.5	
Hepatomegaly	HP:0002240	2	9.5	
Postaxial foot polydactyly	HP:0001830	2	9.5	
Premature tooth eruption	HP:0006288	2	9.5	
Sensorineural hearing impairment	HP:0000407	2	9.5	
Supernumerary flexion creases of the fingers	HP:0040064	2	9.5	
Neoplasm (ganglioneuroma)	HP:0002664	1	4.8	
Macrocephaly, craniosynostosis, hypotonia, cryptorchidism, hypospadias, thyroglossal cyst, renal cyst, nephromegaly, splenomegaly, haemangioma, prominent upper eyelids, strabismus, apnoea, psoriasiform dermatitis, visceromegaly		1	4.8	

**Table 3:** BWSp consensus scoring in 21 patients.

**Clinical Features**			Patients and Consensus BWSp Scoring *										
			**OGS1360**	**OGS1810**	**OGS1380**	**OGS1774**	**OGS1449**	**OGS1584**	**OGS1775**	**OGS2051**	**OGS2052**	**OGS2166**	
			**Score 5**	**Score 5**	**Score 4**	**Score 3**	**Score 4**	**Score 8**	**Score 5**	**Score 6**	**Score 5**	**Score 5**	
Macroglossia	HP:0000158	CF	X	X	X	X	X	X	X	X	X	X	
Omphalocele	HP:0001539	CF						X		X	X	X	
Anterior earlobe creases	HP:0009908	CF								X	X	X	
Overgrowth	HP:0001548	SF			X		X	X	X				
Posterior helix pits	HP0008523	SF						X	X				
Transient hypoglycaemia	HP:0001998	SF			X	X		X		X			
Umbilical hernia	HP:0001537	SF					X		X				
Nevus flammeus	HP:0001052	SF	X	X				X					
Hemihypertrophy	HP:0001528	SF	X	X									
Hepatomegaly	HP:0002240	SF											
Nephromegaly	HP:0000105	SF											
**Clinical Features**			Patients and Consensus BWSp Scoring *										
			**OGS1492**	**OGS1493**	**OGS1494**	**OGS1074**	**OGS542**	**OGS4**	**OGS851**	**OGS145**	**OGS1234**	**OGS491**	**OGS1356**
			**Score 5**	**Score 5**	**Score 5**	**Score 5**	**Score 11**	**Score 6**	**Score 6**	**Score 4**	**Score 6**	**Score 6**	**Score 6**
Macroglossia	HP:0000158	CF	X	X	X	X	X	X	X	X	X	X	X
Omphalocele	HP:0001539	CF	X	X	X		X		X	X	X	X	X
Anterior earlobe creases	HP:0009908	CF	X	X	X						X		X
Overgrowth	HP:0001548	SF				X	X	X				X	
Posterior helix pits	HP0008523	SF	X	X	X	X	X	X					
Transient hypoglycaemia	HP:0001998	SF					X	X	X				X
Umbilical hernia	HP:0001537	SF					X	X	X				
Nevus flammeus	HP:0001052	SF					X				X		
Hemihypertrophy	HP:0001528	SF					X						
Hepatomegaly	HP:0002240	SF				X						X	
Nephromegaly	HP:0000105	SF				X							

* BWSp consensus scoring according to Brioude et al. (2018). CF, cardinal features of BWS; SF, suggestive features of BWS. X indicates the presence of the clinical feature.

## Data Availability

Authors can confirm that all relevant data are included in the article.
